# American Dental Association

**Published:** 1890-03

**Authors:** 


### AMERICAN DENTAL ASSOCIATION.

There seems to be a strong feeling still existing in the minds of the profession that the American Dental Association should meet in the East this year, and at an earlier date than usual, notwithstanding the fact that the question has been once voted upon by the Executive Committee and the officers of the Association. There is a movement on foot to petition the powers that be to make the change. The ground taken by those who are foremost in the movement is that a large number of the members of the Association desire to go abroad, and that if the meeting is held in August, and at Excelsior Springs, they will be unable to attend. There can be no reasonable objection raised against the change in the time and place of meeting of the Association, except that of fickleness, and the evident willingness, on the part of some, to make the American Dental Association secondary to other meetings. It was known last year that, in all probability, the International Medical Congress would be held at its usual time, and the question of conflict in the two dates was discussed, and the time and place of meeting decided upon in the light of that knowledge. It seems to us that then was the time to have raised objections, and not at this late date. Then, again, the Association has been promising for a long time to go to the Southwest, and now that the promise is on the verge of fulfilment, it does not seem just right to vote to go elsewhere. At any rate, if the change is made, it should be with the explicit understanding that it is to return next year to Excelsior Springs, and thus fulfil the promise made to the Western members; and then it can go to Chicago the following year, when the grand memorial meeting is to be held. The Southwest has waited patiently for an opportunity to show its hospitality, and it should not now be deprived of the privilege. Under the circumstances, however, it seems best to make the change asked, as all are, or at least should be, anxious to see the section on dentistry in the Tenth International Medical Congress made as great a success as possible; and if the Western members will allow the change to be made, their magnanimity will be fully appreciated, and the return meeting, next year, will be all the greater success. The meeting could be called for Niagara for the second week in July, immediately following the meeting of the Association of Dental Faculties. The coming meeting of the association will be an important one, as arrangements should be started looking towards a grand meeting in Chicago in 1892. Committees should be appointed and a plan adopted in order to insure that that meeting shall be a success commensurate with the importance of the occasion.

